# Energy Availability as a Neurocognitive Regulator of Endurance Performance: Integrating Metabolic, Perceptual, and Decision-Making Mechanisms—A Narrative Review

**DOI:** 10.3390/sports14040150

**Published:** 2026-04-13

**Authors:** Gerasimos V. Grivas, Walaa Jumah Alkasasbeh

**Affiliations:** 1 Physical Education and Sports, Division of Humanities and Political Sciences, Hellenic Naval Academy, 18539 Piraeus, Greece; 2 Department of Physical Education, School of Sports Sciences, The University of Jordan, Amman 11942, Jordan; walaakasasbeh1991@yahoo.com

**Keywords:** endurance performance, energy availability, low energy availability, perceived exertion, carbohydrate availability, mental fatigue

## Abstract

Endurance performance is regulated through dynamic interactions between physiological capacity, nutritional status, and psychological control processes. While traditional endurance models have emphasized metabolic and cardiorespiratory determinants, growing evidence indicates that energy availability also influences cognitive function, perceived effort, and decision-making during prolonged exercise. This narrative review synthesizes current literature on the interplay between nutritional strategies and psychological regulation in endurance sports, with particular emphasis on low energy availability, carbohydrate availability, mental fatigue, and pacing behavior. Acute and chronic reductions in energy availability are associated not only with endocrine and metabolic disturbances but also with amplified perceived exertion, impaired executive functioning, reduced effort tolerance, and altered risk-related decision-making, even in the absence of overt physiological failure. Carbohydrate availability emerges as a central modulator operating through both peripheral mechanisms (substrate supply and glycogen preservation) and central neurocognitive pathways influencing perception, motivation, and fatigue regulation. Hydration status, caffeine ingestion, and gastrointestinal tolerance further interact with perceptual and cognitive processes to shape real-time pacing and endurance sustainability. Integrating sport nutrition and sport psychology provides a unifying framework for understanding endurance regulation as a multilevel process linking metabolic state to perceptual experience and behavioral decision-making. From an applied perspective, optimizing endurance performance requires maintenance of adequate long-term energy availability, strategic carbohydrate periodization aligned with training demands, and systematic monitoring of perceived effort alongside physiological load. Future research should prioritize interdisciplinary, ecologically valid designs combining metabolic, perceptual, and cognitive measurements, supported by wearable and data-driven technologies capable of capturing real-time endurance regulation. Bridging nutritional and psychological mechanisms within a unified conceptual model offers a stronger scientific basis for improving performance sustainability while safeguarding athlete health in modern endurance sport.

## 1. Introduction

Endurance performance is widely recognized as a complex and multifactorial phenomenon arising from the interaction of physiological, psychological, and environmental factors. Traditional approaches in endurance science have primarily focused on physiological determinants such as maximal oxygen uptake (VO_2max_), lactate thresholds (LT), substrate utilization, and thermoregulatory capacity, which are considered key contributors to performance potential [1,2]. Although these factors define important biological limits, they do not fully explain the variability in endurance performance observed among athletes with comparable physiological profiles.

In recent decades, increasing attention has been directed toward the role of psychological regulation in endurance exercise. Perceived effort, motivation, attentional focus, and cognitive control have been identified as critical determinants influencing the regulation of exercise intensity and the capacity to sustain prolonged effort [3,4]. Contemporary psychobiological and perception-based models propose that endurance performance is governed by continuous integration of afferent physiological feedback, prior experience, and goal-directed cognitive processes rather than by peripheral fatigue alone [5,6]. Accordingly, endurance athletes continuously interpret internal sensory signals, evaluate task demands, and dynamically adjust pacing strategies, particularly under conditions of accumulating fatigue and mental strain [7,8]. These mechanisms highlight the central role of cognitive and perceptual regulation in endurance performance, extending beyond purely peripheral physiological explanations and supporting an integrated psychophysiological framework of exercise tolerance.

Nutrition represents a fundamental, yet often compartmentalized, component within this regulatory framework. Sport nutrition research has traditionally emphasized the metabolic role of nutritional strategies, particularly carbohydrate availability, in supporting energy production and delaying physiological fatigue [9,10]. However, growing evidence suggests that nutritional status also exerts significant effects on brain function, perception of effort, mood, and decision-making processes [11,12]. Consequently, nutritional strategies may influence endurance performance not only through peripheral metabolic pathways but also through central and psychological mechanisms.

Energy availability, defined as the amount of dietary energy remaining for physiological functions after accounting for exercise energy expenditure, has emerged as a critical factor linking nutrition and performance regulation [13]. Acute or chronic states of low energy availability have been associated with impairments in both physical performance and cognitive function, potentially increasing perceived exertion and reducing self-regulatory capacity [14,15]. These effects suggest that endurance performance may be compromised even in the absence of overt physiological dysfunction, through alterations in perceptual and cognitive processes.

Despite these overlapping influences, sport nutrition and sport psychology have often evolved as parallel but relatively disconnected disciplines. Nutritional research has largely focused on optimizing substrate availability, metabolic regulation, and physiological adaptation to training [16,17], whereas sport psychology has concentrated on perceptual, motivational, and cognitive determinants of performance regulation, including perceived exertion, self-regulation, and decision-making processes during prolonged exercise [3,6,7]. This conceptual separation limits a comprehensive understanding of how nutritional strategies interact with psychological regulation to shape endurance performance and pacing behavior [4,18].

An integrative perspective that bridges sport nutrition and sport psychology is therefore warranted. Examining the interactions between energy availability, perceived effort, mental fatigue, and decision-making may provide deeper insight into endurance regulation and the mechanisms underlying pacing strategies [8,19]. Such an interdisciplinary framework has important implications for both theoretical models of endurance performance and applied practice in training and competition [20,21].

Building upon these converging lines of evidence, endurance performance may be more accurately understood within a neurocognitive–metabolic regulatory framework in which energy availability functions as an upstream integrative signal linking physiological capacity with perceptual and executive control processes [3,7,14,15]. These interactions may be partly mediated by neurobiological pathways linking metabolic status to central regulation, including altered brain glucose availability in executive cortical regions, shifts in serotonin–dopamine balance associated with central fatigue, and neuroendocrine signals such as cortisol, leptin, and ghrelin that may influence motivation, perceived effort, and pacing decisions during prolonged exercise [14,15,22]. Within this perspective, insufficient energy availability does not merely constrain substrate supply but may also modulate cortical processing of effort-related signals, influence motivational valuation, and alter pacing-related decision-making before overt peripheral fatigue emerges. Accordingly, energy availability can be conceptualized as a systems-level regulator operating across metabolic stability, perceptual experience, and cognitive control, thereby shaping real-time endurance regulation and long-term performance sustainability. Advancing this integrative view may provide a theoretical basis for unifying sport nutrition and sport psychology within a single model of endurance performance.

The aim of this narrative review is to synthesize current evidence on the interactions between nutritional strategies and psychological regulation in endurance sports, with a particular focus on energy availability, perceived effort, and decision-making processes. By integrating physiological and psychological perspectives, this review seeks to highlight key mechanisms, practical implications, and future research directions within an interdisciplinary framework of endurance performance.

## 2. Energy Availability as a Determinant of Endurance Performance

### 2.1. Concept and Definition of Energy Availability

Energy availability (EA) represents the amount of dietary energy remaining for physiological processes after accounting for the energetic cost of exercise, typically expressed relative to fat-free mass [23]. The concept of EA has become central in contemporary sport nutrition because it emphasizes the energetic constraints imposed by high training loads rather than focusing solely on body mass or total energy balance [23,24]. Importantly, EA is distinct from overall energy balance, as athletes may maintain stable body mass while still experiencing insufficient energy availability to support optimal endocrine, metabolic, and adaptive function [14,15,25].

In endurance sports, EA is highly dynamic, fluctuating across training cycles, competition phases, and recovery periods [23]. When EA is persistently reduced, physiological systems may downregulate processes not essential for immediate survival, thereby compromising training adaptation, recovery, and performance capacity [14,15,23]. Although a value of ~30 kcal·kg^−1^ FFM·day^−1^ has historically been proposed as a reference threshold for low energy availability [23,25], recent Relative Energy Deficiency in Sport (RED-S) conceptual and physiological models indicate that responses to reduced energy availability occur along a continuum influenced by individual moderating factors, rather than a fixed universal cutoff, particularly in male athletes [15].

Adequate EA is therefore essential for maintaining metabolic homeostasis, optimizing endurance adaptations, and sustaining both physical and cognitive performance capacity [14,15,23]. Moreover, EA should not be considered solely a nutritional variable but rather a regulatory signal influencing multiple physiological and central processes, including endocrine function and fatigue-related brain mechanisms [18,24]. Modern models highlight that insufficient EA may compromise the athlete’s ability to adapt to endurance training, particularly when combined with dense training schedules, inadequate recovery, and heightened perceptual demands [26,27].

### 2.2. Low Energy Availability in Endurance Athletes

Low energy availability (LEA) occurs when dietary energy intake is insufficient to meet the combined energetic demands of exercise and the requirements of normal physiological functioning [23,26]. Within the energy availability framework, LEA reflects a state in which the energy remaining after exercise is inadequate to support endocrine, metabolic, and adaptive processes essential for both health and performance [23]. Importantly, LEA may develop even in athletes who maintain stable body mass, as energy availability is distinct from overall energy balance and can exert regulatory effects independent of overt changes in body composition [25]. Endurance athletes are particularly vulnerable to LEA due to high training volumes, elevated daily energy expenditure, dense competition schedules, and, in some cases, intentional dietary restriction aimed at optimizing body composition [23,26]. However, LEA may also arise unintentionally when athletes fail to match energy intake to fluctuating training demands, particularly during periods of increased workload or inadequate recovery nutrition [24,26]. Contemporary evidence indicates that LEA is not uncommon across both elite and recreational endurance populations, with implications for injury risk, training consistency, and long-term performance sustainability [14,15,24,28].

The recognition of LEA has expanded through the development of the RED-S model, which frames low energy availability as a central driver of multisystem impairments affecting both female and male athletes [14,15,24]. The International Olympic Committee consensus statement emphasizes that chronic LEA can lead to widespread physiological disturbances, including endocrine disruption, impaired metabolic regulation, compromised bone health, suppressed immune function, and reduced training responsiveness [14,15]. This framework extends beyond the traditional Female Athlete Triad and highlights LEA as a broad health- and performance-relevant condition in endurance sport settings [23,26]. From a health and injury perspective, LEA has been strongly associated with impaired bone metabolism and an increased risk of bone stress injuries, which are particularly prevalent in endurance athletes exposed to high repetitive mechanical loading [14,15,26]. Evidence from elite distance runners further indicates that LEA-related outcomes may substantially contribute to injury burden and training disruption, thereby limiting endurance development and long-term athletic progression [28]. Such findings underscore the applied importance of adequate energy availability in supporting skeletal health and maintaining training continuity across competitive seasons [24].

Overall, LEA represents a critical issue in endurance sport, with implications that extend across health, injury risk, and performance sustainability [14,15,26]. Early identification of LEA and the implementation of individualized nutritional strategies aligned with training demands are therefore essential for optimizing endurance adaptations and long-term athletic outcomes [23,24].

### 2.3. Acute Versus Chronic Energy Deficits

Distinguishing between acute and chronic reductions in energy availability is essential for understanding their distinct implications for endurance performance, physiological adaptation, and long-term athlete health [14,15,23]. Acute energy deficits typically occur over short time periods, such as during a single prolonged training session, multi-day training block, or competition, when dietary intake does not adequately match the immediate energetic cost of exercise [17,29]. In endurance athletes, these transient deficits are common due to the large energetic demands of prolonged exercise and the practical difficulties of consuming sufficient fuel during training and racing, particularly when carbohydrate intake is suboptimal [17,30]. Acute reductions in energy and carbohydrate availability may lead to immediate impairments in exercise tolerance, earlier onset of fatigue, and reduced capacity to sustain target intensities during prolonged exercise [17,29]. From a metabolic perspective, insufficient carbohydrate intake accelerates muscle glycogen depletion and contributes to performance decline, even when athletes attempt to maintain workload [10]. Because carbohydrate availability remains a primary determinant of endurance performance, contemporary sport nutrition guidelines emphasize appropriate carbohydrate ingestion strategies during training and competition to sustain both physiological capacity and perceptual regulation of effort [6,17,29]. In this context, under-fueling may not only limit substrate supply but also amplify perceived exertion, thereby influencing pacing behavior and effort tolerance during prolonged exercise bouts [6,19]. Repeated acute under-fueling may also compromise training quality, recovery processes, and the ability to sustain high-intensity workloads across a training week [26,29]. Modern endurance nutrition frameworks therefore advocate individualized fueling practices aligned with the demands of key training sessions, often referred to as “fueling for the work required,” highlighting that adequate energy intake is essential for both acute performance and consistent training adaptation [17,30].

In contrast, chronic energy deficits reflect a sustained mismatch between energy intake and expenditure over weeks or months, resulting in persistent low energy availability [23,26]. Chronic LEA is associated with cumulative physiological disruption and is recognized as a primary driver of the RED-S syndrome, with multisystem consequences affecting both health and performance capacity [14,15,24]. Unlike acute deficits, which may be resolved through appropriate recovery nutrition, chronic deficits can produce prolonged maladaptation, impair endocrine and metabolic function, and reduce the athlete’s capacity to tolerate high training loads over time [25,26]. Evidence indicates that chronic LEA is linked to reduced training responsiveness, impaired recovery, increased injury susceptibility, and diminished competitive consistency in endurance athletes [24,26]. Elite distance runners experiencing sustained energetic insufficiency may be particularly vulnerable to bone stress injuries and interruptions in training continuity, which directly constrain long-term endurance development [28].

Overall, acute and chronic energy deficits represent distinct yet interrelated challenges in endurance sport [23,26]. While short-term deficits may transiently impair performance during training or competition, chronic low energy availability poses broader risks to health, physiological adaptation, and performance sustainability [14,15,24], emphasizing the importance of nutritional strategies that support both immediate endurance demands and long-term athlete development [17,30].

### 2.4. Consequences for Physiological Function and Endurance Capacity

Low energy availability and sustained energy deficits exert wide-ranging consequences on physiological function, with direct implications for endurance capacity, recovery, and long-term performance sustainability [24,26]. When energy intake is chronically insufficient, the body undergoes adaptive downregulation of multiple systems in order to conserve energy for immediate survival, often at the expense of training adaptation and athletic performance [14,15,23]. These responses indicate that chronic LEA represents not merely a nutritional issue, but a systemic constraint influencing the athlete’s ability to tolerate training load and achieve optimal endurance adaptations [14,15,24]. One of the primary physiological disturbances associated with chronic LEA involves endocrine disruption [14,15,23]. Reduced energy availability alters hypothalamic–pituitary signaling, leading to suppressed reproductive hormones and broader metabolic dysregulation, which can compromise muscle remodeling, recovery processes, and long-term adaptation [25,26]. These endocrine alterations are particularly relevant in endurance athletes, where the balance between training stress and recovery is critical for maintaining consistent progression and sustained performance development [14,15,24].

Metabolic consequences of LEA include impaired substrate utilization and reduced glycogen restoration, which may limit the athlete’s ability to sustain prolonged workloads or perform repeated high-quality training sessions [24,26]. In endurance sports, carbohydrate availability remains a major determinant of performance, as inadequate glycogen stores accelerate fatigue development and reduce time-to-exhaustion capacity [10,17]. Contemporary sport nutrition guidelines therefore emphasize appropriate fueling strategies not only for acute performance but also for supporting recovery and maintaining training quality across endurance training cycles [29,30]. Chronic under-fueling may thus progressively constrain endurance performance through both metabolic limitations and compromised training adaptation. In addition, injury risk represents another major pathway through which LEA affects endurance performance [14,15,26]. Bone stress injuries are highly prevalent in endurance athletes exposed to repetitive mechanical loading, and evidence from elite distance runners indicates that LEA-related outcomes may substantially increase injury burden and disrupt training continuity [24,28]. Such disruptions can reduce cumulative training exposure, impair recovery, and ultimately limit long-term endurance development and competitive consistency [14,15,26].

Taken together, the physiological consequences of chronic energy deficiency extend beyond transient fatigue and influence the fundamental capacity of the endurance athlete to tolerate training load, recover effectively, and achieve optimal adaptation [14,15,23]. Maintaining adequate energy availability should therefore be viewed as a prerequisite for both athlete health and sustained endurance performance, particularly in sports characterized by high training volumes and prolonged competition demands [24,26].

### 2.5. Effects of Energy Availability on Cognitive and Perceptual Processes

Energy availability plays a critical role not only in physiological capacity for endurance performance but also in the regulation of cognitive and perceptual processes that underpin self-regulation and pacing during prolonged exercise [31]. LEA, whether arising from insufficient dietary intake or excessive energy expenditure, has been shown to negatively affect executive functions such as attention, working memory, decision-making, and inhibitory control [32,33]. These cognitive domains are essential for accurately interpreting internal physiological signals and external task demands during endurance activities. When energy availability is compromised, athletes may experience heightened perceived exertion, reduced attentional focus, and impaired allocation of cognitive resources, which may ultimately lead to suboptimal pacing strategies [34]. From a perceptual perspective, LEA appears to amplify interoceptive sensations associated with fatigue, discomfort, and effort, thereby altering the subjective experience of exercise intensity [35]. This distortion in perception may result in an earlier onset of effort-related aversion and reduced tolerance for sustained workloads, even in the absence of pronounced physiological limitations [36].

In addition, inadequate energy availability may disrupt neuroendocrine and neurotransmitter processes involved in mood regulation, motivation, and cognitive control [37,38], contributing to increased mental fatigue and diminished cognitive resilience during endurance tasks. Collectively, these cognitive and perceptual alterations highlight energy availability as an important determinant of endurance performance, acting through central mechanisms that influence how effort is perceived, regulated, and sustained over time.

## 3. Nutritional Strategies in Endurance Sports

### 3.1. Carbohydrate Availability and Endurance Performance

Carbohydrate availability remains one of the most critical nutritional determinants of endurance performance, as carbohydrates provide a rapidly oxidizable substrate that supports high rates of energy turnover during prolonged exercise [10,16,17,39]. Glycogen stored in skeletal muscle and liver represents a finite resource, and depletion of these stores is closely linked to the development of fatigue and reductions in sustainable exercise intensity [10,17]. Consequently, optimizing carbohydrate availability before and during endurance exercise has long been recognized as a cornerstone of sport nutrition practice [16]. Contemporary guidelines recommend carbohydrate intake strategies aligned with exercise duration and intensity. For events exceeding 2–3 h, exogenous carbohydrate ingestion of ~90–120 g·h^−1^ using multiple transportable carbohydrates (e.g., glucose–fructose combinations) has been shown to enhance endurance performance by maintaining blood glucose availability, increasing exogenous carbohydrate oxidation, and delaying fatigue [16,40,41]. Such fueling strategies are particularly relevant for elite endurance athletes competing at high metabolic intensities, where carbohydrate oxidation capacity is a decisive factor in performance sustainability [16,39].

In addition to race-day fueling, carbohydrate availability is increasingly conceptualized within periodized nutrition frameworks. The approach commonly termed “fueling for the work required” proposes that athletes strategically match carbohydrate intake to the metabolic demands of key training sessions in order to maximize training quality, support recovery, and promote long-term adaptation [27,30,42]. Related discussions on “training low” versus “training high” carbohydrate availability emphasize individualized periodization that balances metabolic signaling objectives with performance requirements [42]. Importantly, real-world endurance fueling is often constrained by gastrointestinal tolerance. Exercise-induced gastrointestinal disturbances may limit carbohydrate intake during prolonged events, underscoring the importance of gut training and individualized fueling strategies [43]. Applied case evidence from elite endurance athletes further highlights that successful carbohydrate planning requires integration of physiological demands, nutritional logistics, and competition-specific constraints [44].

Collectively, carbohydrate availability should be viewed as a dynamic nutritional tool influencing endurance performance through metabolic, gastrointestinal, and central regulatory pathways [16,17,43], with individualized strategies forming a foundation for both acute performance optimization and long-term endurance sustainability [14,15,27].

### 3.2. Fueling Strategies Before, During, and After Exercise

Fueling strategies in endurance sports depend not only on total daily energy intake but also on the timing of nutrient provision across the exercise–recovery cycle. Because metabolic demands fluctuate before, during, and after prolonged activity, endurance athletes must strategically distribute carbohydrate and protein intake to optimize substrate availability, delay fatigue, and enhance recovery [16,29]. Carbohydrate intake in the hours preceding endurance exercise plays a fundamental role in maximizing liver and muscle glycogen stores, thereby supporting sustained workloads. Current recommendations suggest consumption of approximately 1–4 g·kg^−1^ of carbohydrate within 1–4 h before exercise, depending on gastrointestinal tolerance and event demands, while structured carbohydrate-loading protocols remain effective for optimizing glycogen availability prior to prolonged competitions [17,29].

During prolonged endurance exercise, exogenous carbohydrate ingestion helps maintain blood glucose concentrations, reduces reliance on endogenous glycogen stores, and supports higher sustainable exercise intensities. Evidence supports carbohydrate intake rates of ~30–60 g·h^−1^ for moderate-duration sessions and up to ~90 g·h^−1^ or higher for events exceeding 2.5–3 h when multiple transportable carbohydrates are used [17,45]. Practical implementation is often constrained by gastrointestinal tolerance, highlighting the importance of individualized gut-training strategies to improve carbohydrate delivery during competition [6,46].

Post-exercise nutrition is essential for restoring glycogen, promoting muscle repair, and preparing the athlete for subsequent training sessions. Rapid glycogen resynthesis is optimized when carbohydrate is consumed soon after exercise, particularly during the early recovery window [47], while combining carbohydrate with protein intake enhances muscle protein synthesis and supports recovery during high-volume training periods [29,48]. Contemporary endurance nutrition frameworks therefore emphasize individualized and periodized fueling practices that align nutrient timing with session intensity and adaptation goals, supporting both acute performance and long-term endurance sustainability [27,30].

### 3.3. Central and Peripheral Effects of Nutritional Intake

Nutritional intake influences endurance performance through both peripheral metabolic pathways and central regulatory mechanisms [17,18]. Traditionally, sport nutrition research has emphasized the peripheral role of substrate availability, particularly carbohydrate supply, in sustaining oxidative energy production and delaying glycogen depletion during prolonged exercise [10,16]. Inadequate fueling accelerates metabolic fatigue, limits sustainable workloads, and constrains endurance performance capacity, especially when glycogen availability becomes performance-limiting [17,29].

Beyond substrate provision, nutritional intake may also exert central effects that influence fatigue perception and effort tolerance during prolonged exercise [4,18]. Experimental studies demonstrate that carbohydrate sensing in the oral cavity can enhance endurance performance even in the absence of substantial metabolic contribution, suggesting a brain-mediated effect independent of substrate delivery [46]. These findings support the view that nutritional signals may influence endurance performance through neural pathways involved in motivation and fatigue perception [6,49]. More recent neurophysiological evidence has further supported this carbohydrate-sensing mechanism, demonstrating that oral carbohydrate exposure may enhance corticomotor excitability and attenuate the effects of mental fatigue, supporting a central regulatory influence on performance [50]. Functional neurophysiological and neuroimaging approaches suggest that carbohydrate mouth rinsing may influence cortical activation and neural pathways linked to motor output and perceived effort regulation [50]. However, recent systematic reviews and meta-analyses indicate that the ergogenic effects of carbohydrate mouth rinsing are context-dependent, with smaller or inconsistent benefits observed in fed athletes and during prolonged exercise exceeding approximately two hours, where metabolic fuel availability becomes the dominant determinant of endurance performance [51].

Additional nutritional factors may interact with neuroendocrine and neurotransmitter responses that influence mood, alertness, and exercise tolerance [18,32]. Under conditions of low energy availability, central fatigue mechanisms may be amplified, whereas optimal fueling may support both physiological stability and sustained exercise capacity during prolonged workloads [14,15,19].

Collectively, these findings indicate that nutritional strategies operate through integrated peripheral and central pathways, influencing endurance performance via substrate availability, fatigue resistance, and physiological stability during prolonged exercise [17,18].

### 3.4. Nutritional Modulation of Fatigue and Effort Tolerance

Fatigue during endurance exercise arises from multiple physiological mechanisms including substrate depletion, dehydration, thermoregulatory strain, and neuromuscular limitations [18,52]. Nutritional strategies can modulate fatigue development by influencing substrate availability, hydration status, and metabolic stability during prolonged exercise [3,17].

Carbohydrate availability remains one of the most established nutritional determinants of fatigue resistance. Inadequate glycogen stores accelerate fatigue development and impair the athlete’s capacity to sustain target intensities, whereas carbohydrate ingestion during exercise can delay exhaustion by maintaining blood glucose availability and supporting continued energy production [10,17]. Carbohydrate mouth sensing may also contribute to improved exercise tolerance, suggesting a complementary central influence on fatigue perception [46]. Hydration and electrolyte balance further contribute to fatigue modulation, particularly during prolonged exercise in warm environments. Dehydration increases cardiovascular strain, elevates core temperature, and accelerates performance decline, underscoring the importance of fluid and sodium strategies for maintaining endurance capacity under thermal stress [53,54,55]. Caffeine supplementation has also been shown to delay fatigue development during endurance exercise, with evidence demonstrating improved endurance capacity and time-trial performance [56,57]. These effects are attributed to both central nervous system stimulation and altered fatigue perception.

Collectively, endurance nutrition strategies targeting carbohydrates, hydration, and ergogenic aids influence fatigue development through physiological mechanisms that support sustained exercise capacity during prolonged workloads [17,18,52]. From an applied perspective, cognitive–metabolic periodization may represent an additional consideration for endurance training design. Training sessions performed under low energy availability or glycogen-depleted conditions may impair executive function, attentional control, and decision-making processes, potentially increasing perceived effort and reducing endurance performance capacity [3,7,8]. Under these conditions, highly complex tactical or cognitively demanding drills may increase the risk of suboptimal pacing decisions and impaired motor learning, particularly when central fatigue alters perception of effort and task engagement [58]. Consequently, low-carbohydrate or “train-low” sessions may be more appropriate for metabolic adaptation using relatively simple motor tasks. Conversely, sessions performed with adequate carbohydrate availability may be better suited for complex decision-making, pacing strategy development, and cognitively demanding training. Alternatively, controlled exposure to cognitively demanding tasks under low energy availability may be strategically implemented to develop resilience and fatigue resistance, although this approach requires careful progression and individualization [8,59].

## 4. Psychological Regulation in Endurance Exercise

### 4.1. Perceived Effort and Performance Regulation

Rating of Perceived Exertion (RPE) is a well-established psychological factor involved in the regulation of performance during endurance exercise [60]. During prolonged physical activity, athletes do not rely exclusively on objective physiological indicators such as heart rate or oxygen consumption [4]; rather, subjective perceptions of effort play a decisive role in pacing, persistence, and voluntary termination of exercise [61]. RPE reflects the integration of physiological feedback from both central and peripheral systems, including afferent signals from active musculature and the respiratory system, while also being shaped by conscious perceptual processes influenced by motivation, experience, and task expectations [62]. Perception-based models of performance regulation propose that athletes continuously adjust exercise intensity to maintain a sustainable balance between perceived effort and the anticipated duration of the task, thereby optimizing pacing and preventing premature fatigue [6].

During prolonged exercise, performance is regulated through brain-mediated pacing mechanisms that distribute effort in response to accumulating physiological strain [63]. The progressive rise in RPE reflects both physiological and cognitive demands and is closely associated with performance decline or voluntary disengagement when perceived effort approaches maximal tolerable levels [3]. Importantly, psychological interventions such as verbal encouragement, attentional strategies, and cognitive reappraisal have been shown to attenuate perceived effort at a given workload and enhance endurance performance without substantial physiological change [64]. These findings support the view that endurance performance is governed by an integrated psychophysiological regulatory process, with RPE functioning as a central self-regulatory signal that links physiological status, cognitive appraisal, and pacing decisions during prolonged exercise.

### 4.2. Mental Fatigue and Its Impact on Endurance Capacity

Mental fatigue is recognized as a psychobiological state resulting from prolonged engagement in cognitively demanding activities [3] and has emerged as a critical factor influencing endurance performance [7]. While its detrimental effects on cognitive and skilled performance are well established, accumulating evidence from psychobiological models indicates that mental fatigue also impairs endurance capacity. According to the psychobiological model of endurance performance, perception of effort represents the primary determinant of exercise tolerance; therefore, factors that increase perceived exertion are likely to reduce endurance performance [59]. Systematic evidence demonstrates that mental fatigue elevates perceived effort and reduces exercise tolerance despite unchanged physiological responses [8]. Under conditions of mental fatigue, individuals reach their maximal tolerable effort earlier and voluntarily disengage from exercise, even when peripheral physiological capacity remains unaffected [65]. These findings indicate that the limiting effect of mental fatigue is mediated primarily through perceptual and cognitive mechanisms rather than peripheral physiological failure, positioning mental fatigue as a key determinant of endurance performance regulation during prolonged exercise.

### 4.3. Motivation, Self-Regulation, and Attentional Focus

Psychological regulation plays a central role in endurance exercise, where athletes must sustain effort over prolonged periods while continuously managing internal states and external demands [66,67]. Key psychological mechanisms underpinning endurance performance include motivation, self-regulation, and attentional focus, which interact dynamically to influence pacing strategies, perceived exertion, and performance sustainability [68,69,70,71]. Motivation represents a foundational driver of endurance behavior and is commonly conceptualized within Self-Determination Theory, which distinguishes between intrinsic and extrinsic forms of motivation [71]. Athletes characterized by stronger intrinsic motivation, such as enjoyment, personal challenge, and mastery, typically demonstrate greater persistence, adaptive pacing, and resilience to fatigue during endurance tasks, whereas controlled forms of motivation may support short-term compliance but are often associated with higher perceived exertion and reduced long-term adherence [72].

Self-regulation refers to the athlete’s capacity to monitor, evaluate, and adjust thoughts, emotions, and behaviors in pursuit of performance goals [73]. During endurance exercise, self-regulatory processes include goal setting, emotional control, effort regulation, and pacing-related decision-making [70], allowing athletes to align effort output with task demands and remaining physiological reserves and thereby reduce the risk of premature exhaustion [74]. Evidence further suggests that self-regulatory capacity becomes increasingly important as fatigue accumulates, particularly in the later stages of endurance events [75]. Attentional focus also modulates endurance performance by shaping how athletes perceive and respond to internal sensations such as discomfort, breathing, and muscular fatigue [69]. Attentional strategies are commonly classified as associative—focusing on bodily sensations and task-relevant cues—or dissociative—diverting attention away from physical sensations [76]. Experienced endurance athletes often adopt flexible attentional strategies, shifting between associative focus during high-intensity phases and dissociative focus during lower-intensity segments to regulate perceived exertion effectively [77].

Collectively, motivation, self-regulation, and attentional focus operate as an integrated regulatory system that shapes how effort is perceived, tolerated, and strategically deployed over time [69,70]. Athletes with stronger autonomous motivation are more likely to engage in effective self-regulatory behaviors and adopt adaptive attentional strategies, thereby enhancing performance consistency and psychological resilience during prolonged exercise [71,74]. This integrative perspective aligns with contemporary psychobiological models of endurance performance, which emphasize the dynamic interaction between motivational processes, cognitive control, and perception of effort in sustaining goal-directed behavior under fatigue [3,7].

### 4.4. Cognitive Control and Executive Function in Prolonged Exercise

Cognitive control and executive function constitute essential psychological processes underpinning performance during prolonged exercise [31]. These higher-order functions enable athletes to regulate attention, inhibit maladaptive responses, and adapt behavior in accordance with task demands and performance goals under sustained physiological stress [78,79]. In endurance contexts, executive processes are continuously engaged to support effort regulation, pacing decisions, and the maintenance of goal-directed behavior as fatigue develops. Prolonged exercise imposes increasing cognitive demands, particularly on attentional control and inhibitory regulation [80]. Athletes must selectively attend to performance-relevant information, such as pacing cues and breathing patterns [81], while suppressing competing internal signals related to discomfort, fatigue, or disengagement tendencies [82]. As exercise duration increases, the combined effects of physical and mental fatigue may challenge executive functioning, leading to diminished attentional regulation and less adaptive decision-making, which have been associated with suboptimal pacing and premature reductions in effort [78,83].

Contemporary theoretical perspectives suggest that alterations in cognitive control during prolonged exercise should not be interpreted solely as the consequence of depleted cognitive resources but rather as a shift in the prioritization of cognitive processes under increasing physiological and perceptual strain [84,85,86]. Within this framework, effective cognitive control prioritizes task-relevant information and supports sustained engagement with performance goals despite the increasing salience of aversive internal sensations [86].

Overall, cognitive control and executive function play a central role in prolonged exercise performance by enabling adaptive effort regulation and decision-making under fatigue, providing insight into individual variability in endurance capacity and highlighting their importance as psychological determinants of sustained athletic performance.

### 4.5. Psychological Resilience Under Sustained Physiological Stress

Endurance athletes are routinely exposed to sustained physiological stress resulting from prolonged training sessions, high cumulative training loads, and extended competitive demands [87]. Such stress is characterized by persistent metabolic strain, neuromuscular fatigue, and prolonged activation of neuroendocrine systems [88]. Within this context, psychological resilience plays a critical role in enabling athletes to maintain mental stability and functional performance despite continuous physiological challenge [89]. Resilient endurance athletes demonstrate an enhanced capacity to tolerate prolonged discomfort and fatigue while preserving task engagement and psychological balance [90]. Psychological resilience under sustained physiological stress is reflected in adaptive cognitive and emotional processes, with athletes exhibiting higher resilience tending to appraise prolonged physical stress as a manageable challenge rather than an overwhelming threat [91,92,93,94]. This adaptive appraisal supports more effective emotional regulation and sustained attentional focus during extended exercise, facilitating regulation of perceived exertion as physiological strain accumulates and contributing to persistence and effective pacing during endurance tasks [95].

Resilience is also closely linked to self-regulatory and motivational mechanisms that support long-term adaptation to sustained physiological stress [96]. Athletes exhibiting higher psychological resilience are more likely to maintain autonomous motivation and flexible goal adjustment under fatigue, thereby reducing the risk of psychological exhaustion and burnout [97]. Importantly, resilience should be viewed as a dynamic and trainable capacity shaped by repeated exposure to endurance stressors and the development of psychological skills [98]. Strengthening resilience may therefore enhance both performance sustainability and mental well-being in endurance athletes facing chronic physiological demands [99].

Collectively, current evidence indicates that psychological resilience is a key determinant of endurance adaptation under sustained physiological stress, operating through adaptive cognitive appraisal, emotional regulation, and effective control of perceived exertion, and positioning resilience as a central psychological factor supporting long-term performance sustainability and protection against mental fatigue and burnout in endurance athletes.

## 5. Interactions Between Nutrition and Psychological Processes

The integration of sport nutrition and psychological regulation has gained increasing attention in endurance performance research. Athletes regulate exercise intensity not only through peripheral physiological capacity but also through perceptual and cognitive processes that influence effort tolerance and fatigue development [4,70]. Nutritional status may shape the interpretation of physiological signals, perceived exertion, and motivational state during prolonged exercise [6,18]. Emerging psychobiological and integrative models suggest that metabolic availability and central nervous system processes interact to influence fatigue perception and exercise tolerance. Within this framework, energy availability represents a key construct linking metabolic state with perceptual regulation, providing a basis for understanding how fueling strategies influence endurance performance sustainability [14,15,24].

### 5.1. Energy Availability and Perception of Effort

Perception of effort is a key determinant of endurance performance, reflecting the conscious sensation of exercise intensity and influencing exercise tolerance [4,49]. Contemporary models propose that athletes regulate performance through integration of physiological feedback, motivational state, and perceived exertion rather than solely through peripheral fatigue endpoints [6]. Energy availability plays a central role in shaping this perceptual regulation. When dietary intake is insufficient to meet energetic demands, low energy availability may amplify fatigue perception and reduce exercise tolerance [14,15,23]. Under such conditions, athletes may experience elevated exertional sensations and reduced capacity to sustain prolonged workloads even before overt physiological failure occurs [4,18].

Experimental evidence further indicates that cognitive fatigue interacts with perceived exertion pathways, impairing endurance capacity independently of measurable changes in muscle function [3,19]. Collectively, endurance performance emerges from the interaction between physiological energy availability and perceptual regulation of effort, suggesting that adequate fueling may help stabilize perceived exertion and support sustained exercise tolerance [14,15,18].

### 5.2. Carbohydrate Intake and Cognitive Performance

Carbohydrate intake during endurance exercise has traditionally been viewed as a strategy to sustain peripheral energy supply; however, evidence indicates that carbohydrate availability may also influence cognitive function during prolonged exercise. Because the brain relies heavily on glucose as a primary energy substrate, fluctuations in carbohydrate availability may affect attentional focus and fatigue perception [18,32]. Carbohydrate sensing in the oral cavity has been shown to enhance endurance performance even when direct metabolic contribution is minimal, supporting brain-mediated pathways linking carbohydrate availability with exercise tolerance [46]. These effects may be particularly relevant during prolonged endurance tasks where cognitive factors influence sustained effort [6]. Carbohydrate availability may also modulate mental fatigue, which has been shown to impair endurance performance by elevating perceived exertion independently of neuromuscular changes [3,19]. Adequate carbohydrate intake may therefore support cognitive resilience and reduce the subjective burden of prolonged exercise [17,18].

### 5.3. Nutritional Influences on Affective Responses and Mood

Nutrition also influences affective responses and mood during endurance exercise [100]. Adequate intake of carbohydrates, proteins, and essential fatty acids contributes to neurotransmitter synthesis involved in mood regulation, including serotonin and dopamine pathways [101]. In contrast, insufficient energy intake has been associated with increased stress responses, negative affect, and reduced motivation during exercise [102,103,104,105]. Within endurance contexts, adequate nutritional status may support emotional stability and sustained task engagement during prolonged exercise [106,107]. Positive affective responses have been linked with improved exercise tolerance, whereas negative affect may contribute to premature fatigue perception and reduced endurance capacity [107]. These findings suggest that nutritional status influences endurance performance partly through affective regulation.

### 5.4. Nutrition-Related Modulation of Decision-Making During Exercise

Decision-making during endurance exercise reflects continuous adjustment of effort in response to physiological and perceptual signals. Rather than being governed solely by peripheral physiological limitations, endurance performance emerges from dynamic regulation of effort tolerance during prolonged exercise [6,49]. Within this framework, nutritional state shapes the cognitive and perceptual environment in which these decisions occur. Nutritional status may influence this regulatory process by modifying fatigue perception and exercise tolerance. Carbohydrate availability has been shown to influence endurance performance through both metabolic and central pathways, suggesting that nutritional cues contribute to effort regulation during prolonged exercise [46]. Conversely, low energy availability may amplify fatigue perception and reduce sustained exercise capacity [14,15,18]. Mental fatigue provides an additional link between nutrition and exercise regulation, as cognitive fatigue increases perceived exertion and reduces endurance performance independently of neuromuscular function [3,19]. Nutritional strategies that maintain energy availability may therefore support more stable effort regulation during endurance exercise.

### 5.5. Integrative Models Linking Physiological and Psychological Regulation

Contemporary integrative models of endurance performance emphasize interaction between physiological state and psychological regulation [21]. These frameworks propose that performance emerges from continuous integration of metabolic signals, neuroendocrine responses, and perceptual processes [108,109]. Signals related to energy availability and physiological strain are interpreted within higher-order brain networks [61], where they interact with motivational and contextual factors to influence exercise tolerance [110]. Perceived exertion, affective responses, and mental fatigue function as mediators linking physiological stress with behavioral regulation [94]. These mechanisms also account for individual variability in psychological resilience, interoceptive awareness, and self-regulatory capacity, providing a comprehensive explanation for divergent performance outcomes among athletes with comparable physiological profiles [111].

Collectively, these integrative models describe endurance performance as the emergent product of tightly coupled physiological and psychological processes, supporting interdisciplinary approaches that consider both metabolic and perceptual determinants of endurance capacity.

## 6. Decision-Making and Pacing in Endurance Sports

### 6.1. Pacing as an Integrated Cognitive–Physiological Process

Pacing in endurance sports is increasingly conceptualized as an integrated cognitive–physiological process rather than a purely physiological regulation of effort [112]. Contemporary models propose that athletes continuously regulate exercise intensity through dynamic interactions between central nervous system activity and peripheral physiological signals, including afferent feedback related to metabolic strain, thermoregulation, and neuromuscular fatigue [113]. This regulation is mediated by higher-order cognitive functions such as perception of effort, anticipated task duration, prior experience, and goal-directed decision-making [3]. Within the psychobiological model of endurance performance, pacing decisions are consciously adjusted according to the athlete’s interpretation of internal sensations and anticipated remaining demands rather than dictated by fixed physiological thresholds [94,112].

From a decision-making perspective, pacing represents a continuous evaluation of risk and reward under conditions of physiological stress and uncertainty [114]. Athletes integrate real-time sensory feedback with pre-race strategies, environmental constraints, and competitive context to optimize performance while avoiding premature exhaustion [115]. Neurocognitive evidence further indicates that brain regions associated with executive control and interoceptive awareness contribute to pacing regulation [63,116], underscoring the importance of attentional control, self-regulation, and metacognitive monitoring during prolonged exercise [117]. Accordingly, effective pacing reflects not only physiological capacity but also the athlete’s ability to make adaptive decisions under fatigue [118], positioning pacing at the intersection of endurance physiology, sport psychology, and cognitive neuroscience. Overall, pacing in endurance sports should be understood not as a direct expression of physiological capacity but as the outcome of a continuous decision-making process in which internal physiological signals are integrated with higher-order cognitive regulation to guide adaptive effort distribution.

### 6.2. Nutritional Status and Pacing Behavior

Nutritional status plays a critical role in shaping pacing behavior in endurance sports through its direct influence on both physiological capacity and cognitive functioning [40]. Adequate availability of key substrates, particularly carbohydrates, is essential for sustaining energy production and delaying peripheral fatigue, thereby enabling athletes to maintain planned pacing strategies over prolonged durations [119]. Conversely, depleted glycogen stores or insufficient energy intake can accelerate fatigue-related sensory feedback, including heightened perceptions of effort and discomfort that may prompt premature reductions in exercise intensity [120]. From this perspective, nutritional status functions as a physiological constraint that modulates the afferent signals informing pacing decisions and ultimately influences how effort is distributed across an endurance task [121].

Beyond peripheral physiology, nutritional state also shapes pacing behavior through cognitive and perceptual mechanisms [122]. Nutritional insufficiency, dehydration, or hypoglycemia may impair attention, executive control, and decision-making accuracy during prolonged exertion [121], thereby compromising the athlete’s ability to interpret internal sensations, adhere to pre-planned pacing strategies, and adapt to evolving task demands [123]. In contrast, optimal nutritional support promotes cognitive resilience [124], strengthens self-regulatory capacity [125], and stabilizes perception of effort, enabling more consistent and strategically effective pacing patterns.

Accordingly, pacing behavior should be conceptualized as the outcome of interacting nutritional, physiological, and cognitive regulatory processes, underscoring the importance of integrating nutritional considerations into contemporary models of endurance decision-making and performance regulation.

### 6.3. Fatigue Perception, Risk-Taking, and Performance Decline

Perception of fatigue represents a central mechanism linking physiological strain to behavioral regulation and performance outcomes in endurance sports. As fatigue accumulates, athletes experience heightened sensations of effort, discomfort, and reduced control, which directly influence pacing adjustments and strategic decision-making [126,127]. Rather than reflecting peripheral fatigue alone, fatigue perception emerges from the brain’s integrative interpretation of physiological feedback, contextual information, and prior expectations [128]. This subjective appraisal plays a decisive role in signaling when continued effort may threaten task completion, thereby promoting conservative pacing behaviors or reductions in exercise intensity [129]. Consequently, performance decline is often preceded not by absolute physiological failure but by anticipatory regulation driven by perceived fatigue and effort-related decision processes [6].

Fatigue perception also shapes athletes’ risk-taking behavior, influencing how aggressively or conservatively performance is regulated under prolonged stress [7,130]. During earlier stages of endurance tasks, athletes may tolerate higher discomfort and adopt relatively risky pacing strategies to gain competitive advantage [131], whereas increasing internal or external threat signals progressively shift decision-making toward risk-averse regulation aimed at preserving task completion and physiological safety [132]. Cognitive fatigue may further compound this transition by impairing executive control, attentional regulation, and evaluative judgment, thereby increasing the likelihood of suboptimal pacing decisions and accelerated performance decline [133].

Overall, performance deterioration in endurance exercise should be conceptualized as the emergent outcome of interacting perceptual, cognitive, and decision-making processes rather than as a simple consequence of declining physiological capacity alone. This perspective reinforces contemporary psychobiological and integrative models in which perceived fatigue functions as a primary regulator of endurance behavior and pacing stability [3,6,7].

### 6.4. Pacing Literacy and Learned Decision-Making in Endurance Athletes

Pacing literacy refers to the athlete’s acquired capacity to regulate effort effectively across varying temporal, environmental, and competitive demands, integrating physiological knowledge with experiential and cognitive learning processes [134]. Rather than representing an innate ability, pacing competence develops progressively through repeated exposure to endurance tasks, performance feedback, and reflective evaluation of prior outcomes [135,136]. Through this iterative learning process, athletes construct internal predictive models that support anticipation of fatigue development, more accurate interpretation of interoceptive signals, and alignment of effort distribution with event-specific constraints and performance goals [136,137]. Consequently, pacing literacy enables a transition from reactive intensity adjustments toward proactive and strategically regulated decision-making, particularly under conditions of uncertainty or competitive pressure.

Learned decision-making in endurance sports is shaped by the interaction between accumulated experience, self-regulatory capacity, and contextual awareness [69,136]. As experience increases, athletes refine their ability to balance performance-enhancing risk with task-completion safety, selecting pacing strategies that optimize competitive outcomes [130]. This adaptive learning process is reinforced through deliberate practice, feedback-guided reflection, and the development of cognitive skills such as attentional control and metacognitive monitoring, which support flexible recalibration of effort in response to fatigue, environmental stressors, and race dynamics [117,138].

From a long-term athlete-development perspective, effective pacing should therefore be conceptualized as a learned cognitive–behavioral skill embedded within endurance training rather than merely an expression of physiological capacity. This interpretation highlights the importance of structured decision-focused training, competition simulation, and reflective performance analysis in cultivating adaptive pacing behavior and sustained endurance performance [130,135]. In applied settings, pacing literacy may also be developed by combining cognitive pre-fatigue or decision-making challenges with subsequent endurance efforts, allowing athletes to practice pacing regulation under conditions of elevated perceived effort. Such approaches may help simulate competitive scenarios in which cognitive strain and metabolic fatigue interact, requiring athletes to maintain decision-making accuracy and effort distribution. Repeated exposure to these conditions may strengthen adaptive pacing behavior and improve the integration of perceptual feedback with strategic effort regulation across varying metabolic and perceptual states.

### 6.5. Implications for Competitive Endurance Events

Contemporary evidence suggests that performance in competitive endurance events is not determined solely by physiological capacity but is fundamentally shaped by decision-making processes [139], self-regulatory skills [134], and the perception of fatigue within dynamic competitive environments [133]. This perspective underscores the importance of preparing athletes not only physically but also cognitively and psychologically [140], through the development of pacing literacy, accurate interpretation of bodily signals [138], and adaptive responses to competitive and environmental stressors [141]. In head-to-head endurance events, where tactical interactions and uncertainty are inherent, effective pacing and decision-making require a continuous balance between risk-taking and risk-averse strategies [142].

Accordingly, training programs should integrate decision-focused pacing practice, attentional control strategies, and context-specific feedback to enhance athletes’ capacity to sustain performance, avoid premature performance decline, and respond adaptively to evolving race dynamics. Collectively, these implications support a holistic approach to endurance competition preparation that emphasizes the integration of psychophysiological regulation and learned decision-making as central determinants of competitive success.

## 7. Practical Implications for Athletes, Coaches, and Practitioners

### 7.1. Translating Integrative Evidence into Applied Practice

The translation of integrative evidence from sport nutrition and sport psychology into applied endurance practice requires an athlete-centered approach that considers both physiological fueling demands and perceptual regulation during prolonged exercise. Endurance performance is influenced not only by metabolic substrate availability but also by the interpretation of fatigue-related sensations and the regulation of effort over time [7,31]. Accordingly, fueling strategies should be conceptualized as interventions that support both peripheral energy supply and exercise tolerance during sustained workloads. From a nutritional perspective, carbohydrate intake should be matched to event duration, intensity, and individual tolerance, while real-world endurance settings highlight that successful fueling is often constrained by gastrointestinal robustness and athlete-specific race demands. Field-based evidence from competitive endurance events indicates that nutrition-related gastrointestinal distress remains a common limitation, reinforcing the need for individualized gut-training and practical carbohydrate delivery strategies [43,143]. Elite case studies further demonstrate that carbohydrate periodization across training cycles can enhance performance sustainability when integrated with competition-specific preparation [44].

Translating evidence into practice also requires alignment with training structure and recovery demands [144]. Contemporary endurance frameworks emphasize that adaptive development depends on coordinated load management, recovery support, and strategically timed fueling rather than uniform dietary prescriptions across sessions [20,145,146]. Collectively, these considerations highlight that applied endurance optimization requires integration of physiological demands, nutritional planning, and real-world competition constraints.

### 7.2. Coordinating Nutritional Strategies with Psychological Skills Training

Effective endurance performance also depends on coordination between nutritional planning and psychological skills training [147]. Nutritional strategies that ensure adequate energy availability and stable substrate supply may reduce excessive perceptions of effort and mental fatigue, thereby supporting the application of psychological skills such as attentional control, goal setting, and self-regulation during prolonged exercise [121]. When athletes are adequately fueled, they may be better positioned to interpret physiological signals, maintain cognitive focus, and sustain effort during demanding endurance tasks [49]. From a practical standpoint, coordinated interventions that align fueling practices with psychological training may enhance performance consistency and resilience under competitive stress [148]. Nutrition plans that stabilize blood glucose availability and delay fatigue may complement mental skills targeting attentional control and emotional regulation, particularly during prolonged or high-intensity endurance efforts [149].

Integrating nutritional education within psychological skills training therefore strengthens the interaction between physiological state and mental regulation, supporting performance optimization and long-term training sustainability.

### 7.3. Monitoring Perceived Effort Alongside Fueling Strategies

Effective endurance preparation requires integration of physiological fueling strategies with systematic monitoring of perceptual responses during training and competition. Perceived effort represents a central regulatory signal reflecting the interaction between metabolic strain, neuromuscular fatigue, and cognitive appraisal during prolonged exercise [3,6,150]. Combining nutritional planning with assessment of perceived exertion provides a practical framework for optimizing both training adaptation and competition performance, particularly in endurance contexts where internal load monitoring complements physiological indicators of readiness [17,18,144].

In applied sport settings, RPE has emerged as a widely used tool for quantifying internal training load and monitoring athlete readiness. Session-RPE methodologies enable coaches to capture cumulative physiological and psychological stress across training cycles, offering a practical approach to workload regulation and recovery management [144,151]. Perceptual monitoring may detect maladaptive fatigue or insufficient recovery earlier than external performance metrics, making it particularly valuable when nutritional status fluctuates across training phases [18,152].

Fueling strategies can directly influence perceived effort by modulating substrate availability, hydration status, and fatigue-related responses. Adequate carbohydrate intake during endurance exercise has been shown to attenuate increases in perceived exertion and sustain exercise tolerance, whereas dehydration and low energy availability are associated with elevated effort perception and reduced endurance capacity [17,54]. Integrating RPE monitoring with individualized fueling plans therefore allows practitioners to adjust carbohydrate intake, hydration strategies, and recovery nutrition according to athlete responses during both training and competition [118,146].

From an applied monitoring perspective, practitioners may also benefit from triangulating perceptual responses with physiological and behavioral indicators to better distinguish metabolic fatigue related to under-fueling from cognitive fatigue associated with mental or attentional overload. Integrating RPE monitoring with fueling records, heart rate variability, and emerging wearable or digital readiness tools may support a more individualized interpretation of athlete readiness during training and competition. This integrative approach may help determine whether elevations in perceived effort primarily reflect metabolic constraints or cognitive strain, thereby informing more targeted adjustments in fueling, recovery, and training structure in field-based endurance settings.

### 7.4. Practical Recommendations for Training and Competition

Practical endurance preparation should integrate nutritional planning, perceptual monitoring, and training structure to support sustained performance. Carbohydrate intake should be aligned with exercise intensity, duration, and individual gastrointestinal tolerance to support glycogen availability and sustained work rate during prolonged exercise [16,29]. Repeated rehearsal of race-specific fueling strategies during training enhances gastrointestinal robustness and improves reliability of nutritional execution in competition settings [41]. Incorporating perceptual monitoring into daily training practice allows coaches to detect early signs of excessive fatigue, under-fueling, or inadequate recovery, facilitating adaptive adjustments in workload and nutritional support [144,151]. Hydration planning should also be individualized according to environmental stress and inter-individual variability, as dehydration-related physiological strain may accelerate fatigue development during endurance exercise [54].

Sustaining endurance development over time further depends on maintaining adequate long-term energy availability, structured recovery nutrition, and coherent training periodization, as chronic energetic insufficiency may compromise health, cognitive resilience, and training responsiveness [14,15,20].

From a methodological perspective, practitioners may also consider adjusting training complexity according to energy availability, as reduced carbohydrate availability may influence executive control, decision-making, and pacing behavior during prolonged exercise. Integrating perceptual monitoring with individualized fueling strategies may therefore help distinguish between metabolic fatigue related to under-fueling and cognitive fatigue associated with attentional overload, supporting more informed training and competition decisions.

**Figure 1:**
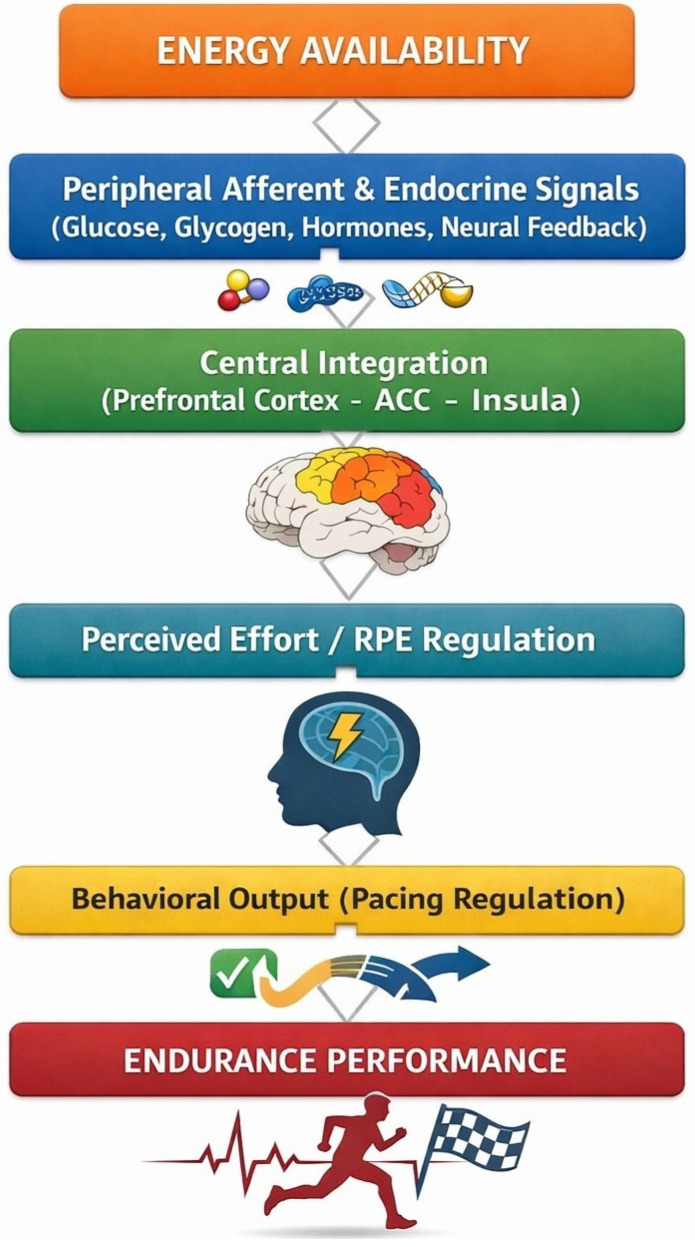
Mechanistic cascade linking peripheral energetic disturbances, central processing, and behavioral regulation of pacing during endurance exercise.

Collectively, endurance optimization should be conceptualized as an interdisciplinary process integrating individualized nutrition, perceptual monitoring, and structured training approaches to support both acute competitive performance and long-term athletic sustainability. Table 1 summarizes the mechanistic relationships between nutritional strategies, perceptual regulation, and endurance performance, while Figure 1 illustrates a mechanistic cascade linking peripheral energetic disturbances, afferent and endocrine signaling, central processing regions, and behavioral regulation of effort and pacing.

## 8. Future Directions

Future research should further investigate the interaction between energy availability, cognitive regulation, and endurance performance using integrative and ecologically valid approaches. Studies incorporating concurrent assessment of metabolic variables, perceptual responses, and executive function during prolonged exercise may clarify how nutritional status influences pacing decisions and fatigue tolerance. Longitudinal and training-intervention designs are also needed to determine causal relationships between chronic energy availability, cognitive resilience, and endurance adaptation. In addition, field-based monitoring and individualized analytical frameworks may improve understanding of within-athlete variability across training and competition. Emerging technologies, including wearable sensors and digital training platforms, may facilitate real-time integration of physiological, perceptual, and nutritional data, supporting personalized endurance regulation and performance optimization.

## 9. Limitations

This narrative review has several limitations that should be acknowledged. First, as a narrative synthesis, the selection and interpretation of evidence may be influenced by conceptual emphasis rather than systematic inclusion criteria, which may limit reproducibility compared with systematic reviews or meta-analyses. Second, the integration of physiological, nutritional, and psychological literature spans heterogeneous methodologies, populations, and outcome measures, potentially constraining direct causal inference. Third, much of the available evidence is derived from laboratory-based protocols with limited ecological validity relative to real-world endurance competition. Finally, empirical validation of the proposed neurocognitive–metabolic framework requires future longitudinal, field-based, and experimentally controlled investigations that simultaneously assess energy availability, perceptual regulation, and decision-making processes. Accordingly, the present review should be interpreted as a conceptual and integrative contribution intended to guide future hypothesis-driven research rather than provide definitive causal conclusions.

## 10. Conclusions

Endurance performance can be conceptualized as a neurocognitive–metabolic regulatory process emerging from the interaction between physiological capacity, nutritional status, and psychological regulation. This narrative review highlights that energy availability influences not only metabolic and endocrine function but also perception of effort, cognitive control, and pacing decisions during prolonged exercise. Insufficient energy availability may amplify perceived exertion, impair executive functioning, and reduce effort tolerance, even in the absence of overt physiological failure. Carbohydrate availability, hydration, and other nutritional strategies therefore shape endurance performance through both peripheral and central pathways interacting with motivational, perceptual, and decision-making processes. Accordingly, endurance optimization should integrate individualized fueling strategies, perceptual monitoring, and pacing-related decision training. Future research should adopt interdisciplinary and ecologically valid approaches to further clarify how nutritional and psychological factors interact to regulate endurance performance.

## Figures and Tables

**Table 1:** Mechanistic links between nutritional factors, central regulation, and pacing behavior in endurance exercise.

Nutritional Factor	Peripheral Physiological Signal	Central Processing Regions	Perceptual/Behavioral Outcome	Implications for Pacing and Performance
Low energy availability	Reduced metabolic substrate availability, endocrine disruption	Hypothalamus, limbic system, prefrontal cortex	Increased fatigue perception, reduced motivation	Conservative pacing strategy and reduced training tolerance
Low carbohydrate availability	Decreased glucose sensing, altered afferent metabolic feedback	Insula, anterior cingulate cortex	Increased RPE, reduced effort tolerance	Earlier pacing downregulation and performance decline
Dehydration	Increased plasma osmolality, cardiovascular strain	Hypothalamus, insular cortex	Elevated perceived strain and thermal discomfort	Reduced work rate and heat-related pacing adjustments
Caffeine and ergogenic aids	Adenosine receptor antagonism, increased CNS activation	Prefrontal cortex, motor cortex	Reduced perceived exertion, enhanced alertness	Improved pacing stability and time-trial performance

## Data Availability

No new data were created or analyzed in this study. Data sharing is not applicable to this article.
